# A Novel Case of Indolent T‐Lymphoblastic Proliferation in Metastatic High‐Grade Serous Adenocarcinoma

**DOI:** 10.1111/ijlh.14556

**Published:** 2025-09-10

**Authors:** Mia Dahl Sørensen, Anja Ør Knudsen, Michael Boe Møller

**Affiliations:** ^1^ Department of Pathology Odense University Hospital Odense Denmark; ^2^ Department of Clinical Research University of Southern Denmark Odense Denmark; ^3^ Department of Oncology Odense University Hospital Odense Denmark

**Keywords:** flow cytometry, indolent T‐lymphoblastic proliferation, leukemia, lymphoma, T‐lymphoblast

1

A 63‐year‐old woman presented with lower extremity swelling and skin thickening of the breasts. Imaging demonstrated generalized lymphadenopathy above and below the diaphragm, hypermetabolic lesions in the left ovary and both breasts, and findings suggestive of peritoneal carcinomatosis. Serum cancer antigen 125 (CA‐125) was elevated at 290 U/mL (reference range: < 35 U/mL). On suspicion of lymphoma or metastatic carcinoma, an excisional inguinal lymph node biopsy was performed. Histological evaluation showed diffuse infiltration by pleomorphic epithelial tumor cells (Figure 1A). Immunohistochemistry demonstrated tumor cells positive for CK7, PAX8, and WT1, with strong and diffuse expression of p53 and p16 (Figure 1B–F). The morphological and immunophenotypic profile was consistent with metastatic high‐grade serous adenocarcinoma, arising from the ovary, fallopian tube, or peritoneum. The stroma was rich in small‐ to medium‐sized tumor‐infiltrating lymphocytes (Figure 1G). Within this infiltrate, a significant subset of lymphocytes displayed blastoid nuclei without overt atypia (Figure 1G, *insert*). Immunohistochemical staining identified these cells as CD3^+^ T‐cells with a precursor immunophenotype, expressing terminal deoxynucleotidyl transferase (TdT), CD1a, and CD10 (Figure 1H–K). Flow cytometric analysis confirmed the presence of an abnormal T‐cell population comprising 27% of the analyzed cells. The cells expressed cytoplasmic CD3 (Figure 2A), TdT (Figure 2A), CD1a (Figure 2B), dim CD99, CD38, dim CD45, as well as CD10, dim CD5, dim CD7, CD2, and co‐expressed CD4 and CD8 (Figure 2C–E). They were predominantly negative for surface CD3 (Figure 2B) and negative for CD34, CD56, CD30, CD279, CD79b, T‐cell receptor (TCR) gamma/delta, and B‐cell markers. The cells thus showed a maturation profile of normal precursor thymocytes of cortical type. Polymerase chain reaction (PCR) testing for the TCR genes revealed no clonal rearrangement, and flow cytometric analysis performed on a concurrent bone marrow aspirate ruled out marrow involvement. Collectively, the work‐up supported the presence of an indolent T‐lymphoblastic proliferation (iT‐LBP) in association with an inguinal lymph node metastasis from a high‐grade serous adenocarcinoma. At diagnosis, the patient had FIGO stage IV ovarian cancer and began neoadjuvant chemotherapy with paclitaxel and carboplatin. Due to persistent extra‐abdominal disease, she was not eligible for interval debulking surgery, and treatment was transitioned to bevacizumab and olaparib. She has since maintained stable disease on olaparib, with normalized CA‐125 and no progressive lymphadenopathy.

**FIGURE 1:**
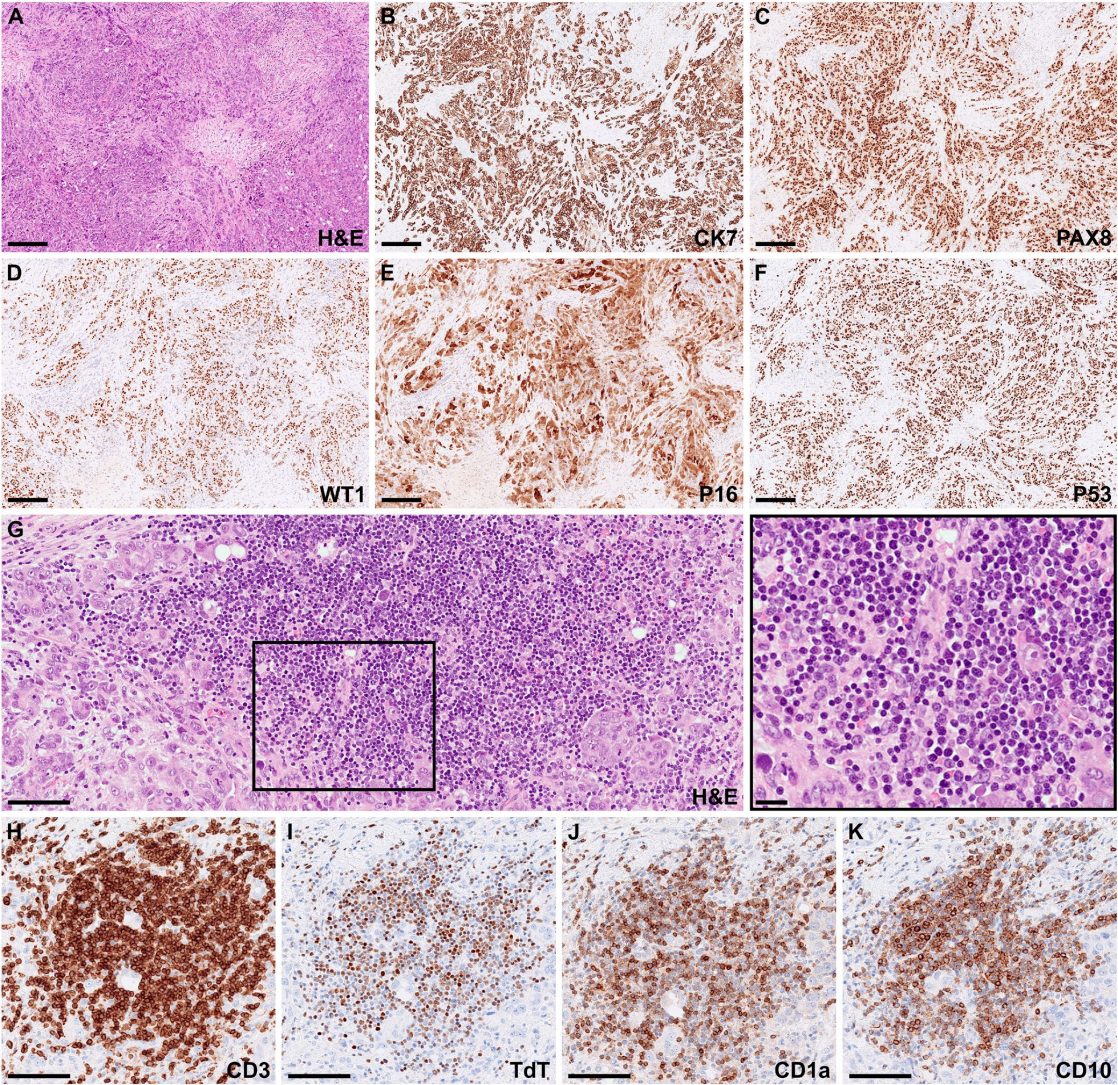
(A–F) Lymph node section showed complete effacement of normal architecture due to diffuse infiltration of pleomorphic epithelial tumor cells. The tumor cells were positive for CK7, PAX8, WT1, p16, and p53, consistent with metastasis from a high‐grade serous adenocarcinoma. (G) Dense clusters of lymphocytes were observed in close association with the epithelial tumor cells, and a large subset of these lymphocytes exhibited blastoid morphology (*insert*). (H–K) The blastoid cells displayed immunopositivity for CD3 (H), TdT (I), CD1a (J), and CD10 (K), resembling a precursor T‐cell phenotype. Scale bar: 250 μm (A–F) or 100 μm (G–K).

**FIGURE 2:**
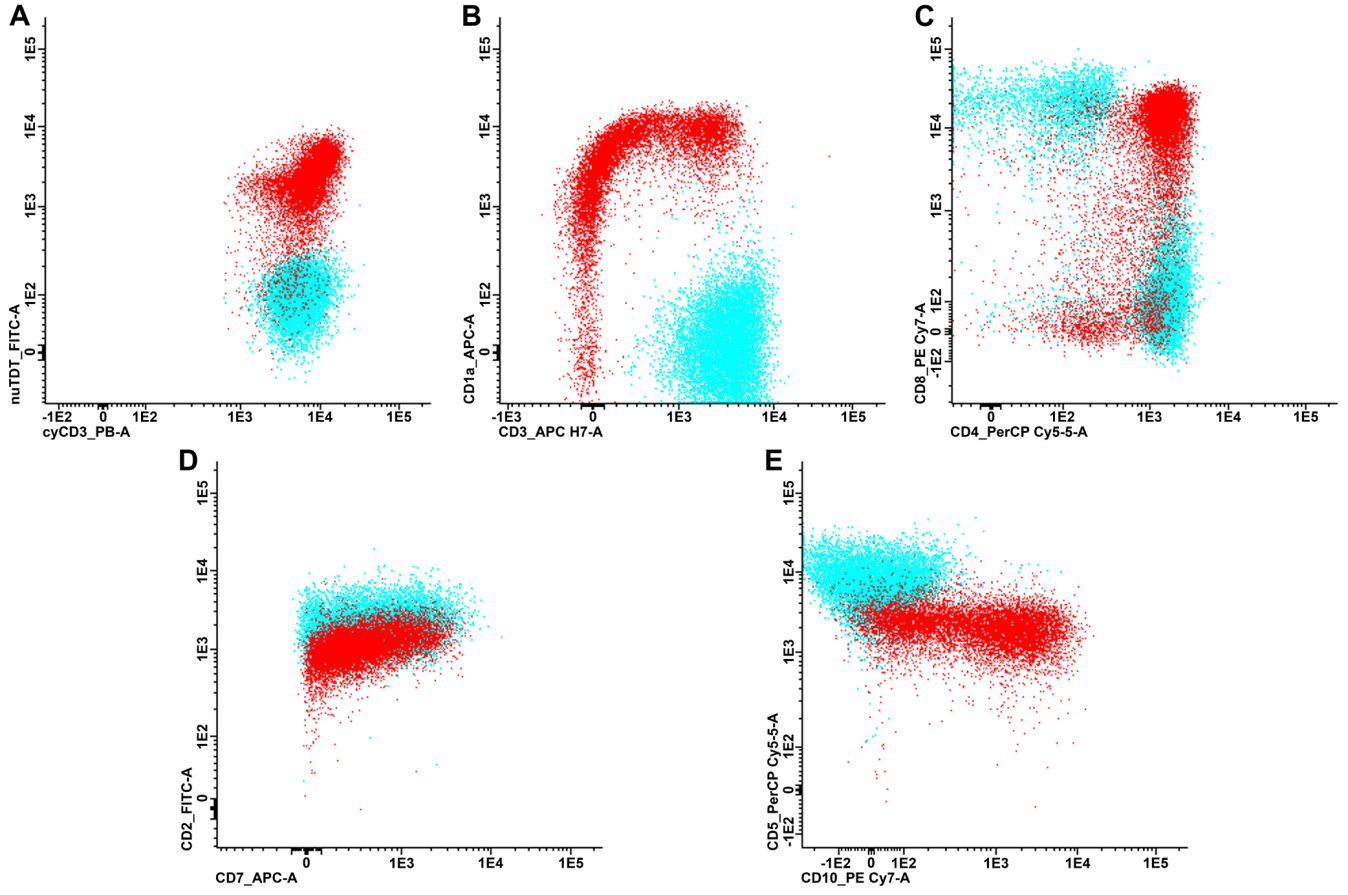
Immunophenotype of the abnormal immature T‐cells (red) compared to normal mature T‐cells (blue). (A–C) Scatter plots show an abnormal population of T‐cells that express cytoplasmic CD3 (A), TdT (A), CD1a (B) and co‐express CD4 and CD8 (C), while they were predominantly surface CD3 negative (B). (D, E) They showed normal expression of CD2 (D) and abnormal expression of CD10 (E), while CD7 (D) and CD5 (E) appeared dim relative to normal mature T‐cells.

iT‐LBP was included as a separate entity in the 5th edition WHO classification of Hematolymphoid Tumors and is defined as confluent groups of non‐clonal T‐lymphoblastic cells without atypia and with an immunophenotype consistent with that of normal T‐lymphoblastic cells expressing CD3 and TdT [1]. iT‐LBP is a clinically indolent disease and considered a rare entity, mostly detected incidentally alongside other pathologies, including hepatocellular carcinoma, Castleman disease, and peripheral T‐cell lymphoma [2]. To our knowledge, however, iT‐LBP has never been reported in association with high‐grade serous adenocarcinoma. iT‐LBP can be diagnostically challenging due to its immunophenotypic overlap with T‐lymphoblastic lymphoma/leukemia, especially as expression of usual T‐cell markers such as CD2, CD5, and CD7 may be reduced [3]. Awareness of this entity is, thus, imperative to ensure a careful diagnostic process and avoid overdiagnosis.

## Author Contributions

Conceptualization: M.D.S. and M.B.M. Data acquisition and interpretation: M.D.S., A.Ø.K., and M.B.M. Drafting of manuscript: M.D.S. All authors have read and approved the final manuscript.

## Ethics Statement

The work was approved by the Region of Southern Denmark's record of data processing activities, and informed verbal and written consent was obtained from the patient.

## Conflicts of Interest

The authors declare no conflicts of interest.

## Data Availability

The data that support the findings of this study are available on request from the corresponding author. The data are not publicly available due to privacy or ethical restrictions.
